# TRPC6 Channels Are Required for Proliferation, Migration and Invasion of Breast Cancer Cell Lines by Modulation of Orai1 and Orai3 Surface Exposure

**DOI:** 10.3390/cancers10090331

**Published:** 2018-09-14

**Authors:** Isaac Jardin, Raquel Diez-Bello, Jose J. Lopez, Pedro C. Redondo, Ginés M. Salido, Tarik Smani, Juan A. Rosado

**Affiliations:** 1 Cellular Physiology Research Group, Department of Physiology, Institute of Molecular Pathology Biomarkers, University of Extremadura, 10003 Caceres, Spain; raqueldiez@unex.es (R.D.-B.); jjlopez@unex.es (J.J.L.); pcr@unex.es (P.C.R.); gsalido@unex.es (G.M.S.); 2 Department of Medical Physiology and Biophysic, Institute of Biomedicine of Sevilla, 41013 Sevilla, Spain; tasmani@us.es

**Keywords:** TRPC6, Orai1, Orai3, store-operated calcium entry, MCF7, MDA-MB-231

## Abstract

Transient receptor potential channels convey signaling information from a number of stimuli to a wide variety of cellular functions, mainly by inducing changes in cytosolic Ca^2+^ concentration. Different members of the TRPC, TRPM and TRPV subfamilies have been reported to play a role in tumorigenesis. Here we show that the estrogen receptor positive and triple negative breast cancer cell lines, MCF7 and MDA-MB-231, respectively, exhibit enhanced expression of the TRPC6 channel as compared to the non-tumoral MCF10A cell line. In vitro TRPC6 knockdown using shRNA impaired MCF7 and MDA-MB-231 cell proliferation, migration and invasion detected by BrdU incorporation, wound healing and Boyden chamber assays, respectively. Using RNAi-mediated TRPC6 silencing as well as overexpression of the pore-dead dominant-negative TRPC6 mutant we have found that TRPC6 plays a relevant role in the activation of store-operated Ca^2+^ entry in the breast cancer cell lines but not in non-tumoral breast cells. Finally, we have found that TRPC6 interacts with Orai1 and Orai3 in MCF7 and MDA-MB-231 cells and is required for the translocation of Orai1 and Orai3 to the plasma membrane in MDA-MB-231 and MCF7 cells, respectively, upon Ca^2+^ store depletion. These findings introduce a novel mechanism for the modulation of Ca^2+^ influx and the development of different cancer hallmarks in breast cancer cells.

## 1. Introduction 

Breast cancer is among the leading causes of cancer death in women worldwide, accounting for about 25% of all diagnosed female cancers [1]. Breast cancer cells are characterized by a high proliferation rate, resistance to programmed cell death, and increased capability to migrate and invade surrounding tissues [2]. These hallmarks can develop through different mechanisms that lead to the onset and progression of breast cancer, among them the alteration in the PI3K pathway [3], abnormal activation of the MAPK signaling [4] or anomalous intracellular Ca^2+^ signaling [5].

Cytosolic free-Ca^2+^ concentration is a crucial factor for a variety of cellular processes [6] and a number of genes encoding ion channels have been found among those altered in cancer cells [7]. There is a growing body of evidence supporting the relevant role of ion channels, and particularly Ca^2+^ channels, in the mechanisms underlying cell growth and proliferation, migration, apoptosis resistance and angiogenesis in cancer cells. Among the Ca^2+^ channels in cancer cells, Orai1, the pore-forming subunit of the Ca^2+^ release-activated Ca^2+^ (CRAC) channel [8,9], which is the best characterized store-operated Ca^2+^ channel, has been found to be overexpressed in the human cancer cells investigated, including breast cancer [10], melanoma [11], clear cell renal carcinoma [12] and non-small cell lung carcinoma [13], except in prostate cancer cells, whose expression has been reported to be reduced as compared to normal tissue [14]. The information concerning the Orai1 homologs Orai2 and Orai3 is rather scarce but Orai2 has been found to be overexpressed in parathyroid adenoma [15] and acute myeloid leukemia cells [16], while Orai3 is overexpressed in estrogen receptor-expressing (ER^+^) breast cancer cell lines [17] and in prostate cancer tissue specimens obtained from resection surgeries as compared to noncancerous tissue [18].

On the other hand, transient receptor potential (TRP) channels, especially certain members of the TRPC, TRPM and TRPV subfamilies, have also been reported to play a relevant role in the progression of different types of cancer. Among them, TRPV6 is overexpressed in a number of cancer cell types and participates in the progression of prostate cancer [19], acquiring its oncogenic potential via Orai1/TRPC1-dependent translocation to the plasma membrane [20]. TRPM8 regulates the motility of a variety of cancer cells including oral squamous carcinoma, lung cancer or prostate cancer cells [21], where its plasma membrane localization and tumorigenic potential are regulated by TRP channel-associated factors [22]. Studies concerning TRPC subfamily members have mainly focused on TRPC1, whose involvement in tumorigenesis varies depending on the stage and type of cancer considered [21,23]. TRPC6 has been reported to play a relevant role in the proliferation of gastric [24], prostate [25], esophageal squamous cell carcinoma [26] and hepatome cells [27]. Furthermore, TRPC6 is required for migration and invasion of hepatocellular carcinoma cells [28]. TRPC6 channels have been shown to be overexpressed in human breast ductal adenocarcinoma compared to non-tumoral tissue [29,30] and both, TRPC3 and TRPC6, have been reported to be significantly up-regulated in breast cancer biopsies compared to normal tissue [31]; however, the molecular basis of the functional role of TRPC6 in breast cancer cells and its involvement in the cancer hallmarks remains unclear.

Here we show that TRPC6 is required for proliferation, migration and invasion of the ER^+^ cell line MCF7 and the triple negative MDA-MB-231 cell line. Silencing TRPC6 protein expression, as well as overexpression of a pore-dead dominant-negative TRPC6 mutant has revealed that TRPC6 plays an important role in the activation of store-operated Ca^2+^ entry (SOCE) in both MCF7 and MDA-MB-231 cell lines, which is likely mediated by the role of TRPC6 in the translocation to the plasma membrane of Orai3 or Orai1, respectively, in the cell lines investigated.

## 2. Results

### 2.1. TRPC6 Is Overexpressed in MCF7 and MDA-MB-231 Breast Cancer Cell Lines and is Required for Breast Cancer Cell Proliferation, Migration and Invasion

**Figure 1:**
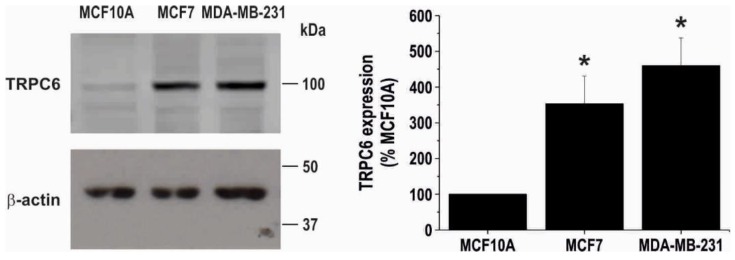
Cellular expression of TRPC6 in non-tumoral and breast cancer cell lines. MCF10A, MCF7 and MDA-MB-231 cells were lysed and subjected to Western blotting with anti-TRPC6 antibody, followed by reprobing with anti-β-actin antibody for protein loading control. Bar graphs represent TRPC6 expression normalized to the β-actin content and expressed as percentage of the TRPC6 expression in non-tumoral MCF10A cells. Molecular masses indicated on the right were determined using molecular-mass markers run in the same gel. * *p* < 0.05 compared to TRPC6 expression in MCF10A cells.

Consistent with the previous study by Aydar and coworkers [31], Western blot analysis of whole cell lysates from the non-tumoral breast MCF10A cell line, the ER^+^ and triple negative breast cancer cell lines MCF7 and MDA-MB-231, respectively, with a specific anti-human TRPC6 antibody revealed that the expression of this protein is relatively low in the non-tumoral cell line (Figure 1). Furthermore, TRPC6 expression in the MCF7 and MDA-MB-231 cell lines is significantly greater (approximately 350 and 460%, respectively) than in non-tumoral cells. TRPC6 expression in the different cell lines, normalized to the β-actin content and expressed as percentage of the expression level in MCF10A, is shown in Figure 1 (bar graphs; n = 6).

We have further explored the involvement of TRPC6 in the ability of MCF10A, MCF7 and MDA-MB-231 to proliferate. To address this issue, cells transfected with shTRPC6 or shRNA control vector (shRNAcv), were subjected to the BrdU cell proliferation assay.

**Figure 2:**
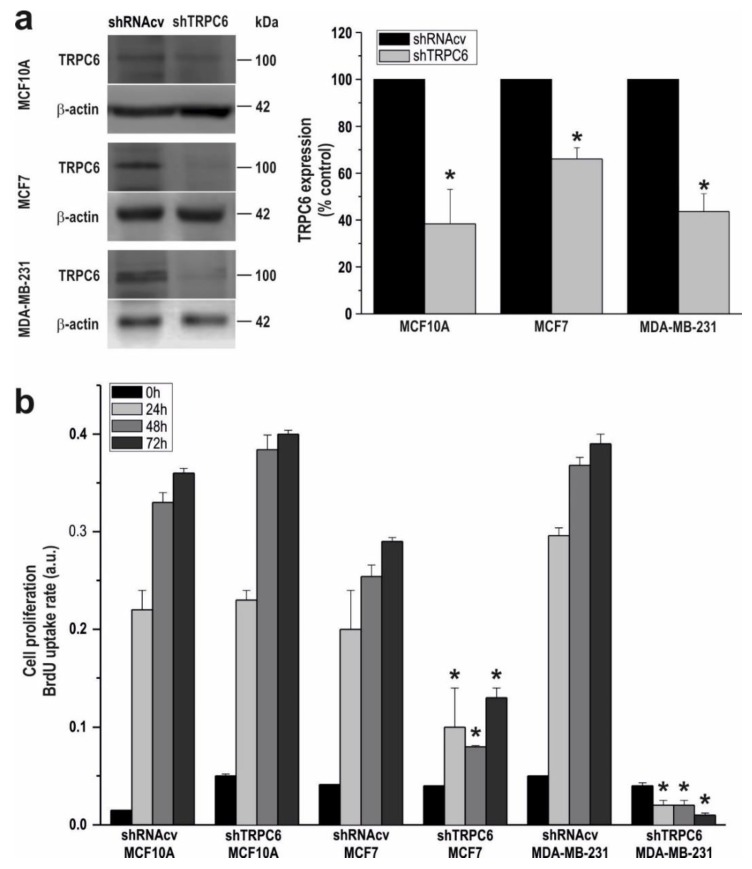
TRPC6 expression is required for MCF7 and MDA-MB-231 cell proliferation. (**a**) MCF10A, MCF7 and MDA-MB-231 cells were transfected with shTRPC6 or shRNA control vector (shRNAcv), as indicated. After 48h cells were lysed and subjected to Western blotting with anti-TRPC6 antibody, followed by reprobing with anti-β-actin antibody for protein loading control. Molecular masses indicated on the right were determined using molecular-mass markers run in the same gel. (**b**) MCF10A, MCF7 and MDA-MB-231 cells were transfected with shTRPC6 or scramble plasmid and 48 h later cell proliferation was assessed for a further 24, 48 and 72 h using the BrdU cell proliferation assay kit, as described in the Material and Methods. Bar graphs represent cell proliferation 0, 24, 48 and 72 h after cell transfection, presented as BrdU uptake rate. * *p* < 0.05 compared to the corresponding control (cells transfected with shRNAcv).

As shown in Figure 2a, cell transfection with shTRPC6 significantly attenuated TRPC6 expression in MCF10A, MCF7 and MDA-MB-231 cells (*p* < 0.05; n = 6). Next, we explored the effect of transfection with shTRPC6 in cell proliferation in the three cell lines. Forty-eight hours after transfection (time = 0 h), as well as 24, 48 and 72 h later, cell proliferation was assessed. As expected, the shTRPC6 was without effect in MCF10A proliferation, which is consistent with the low native TRPC6 expression and indicates a lack of effect of shTRPC6 in cell proliferation in this cell line (Figure 2b; n = 6). Interestingly, silencing TRPC6 protein expression significantly attenuated MCF7 and MDA-MB-231 cell proliferation at all the times investigated as compared to cells transfected with shRNAcv (Figure 2b; *p* < 0.05; n = 4). Therefore, our observations reveal that TRPC6 is essential for ER^+^ and triple negative breast cancer cell proliferation.

**Figure 3:**
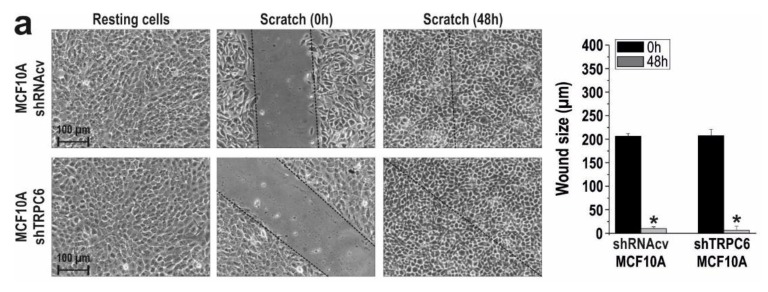

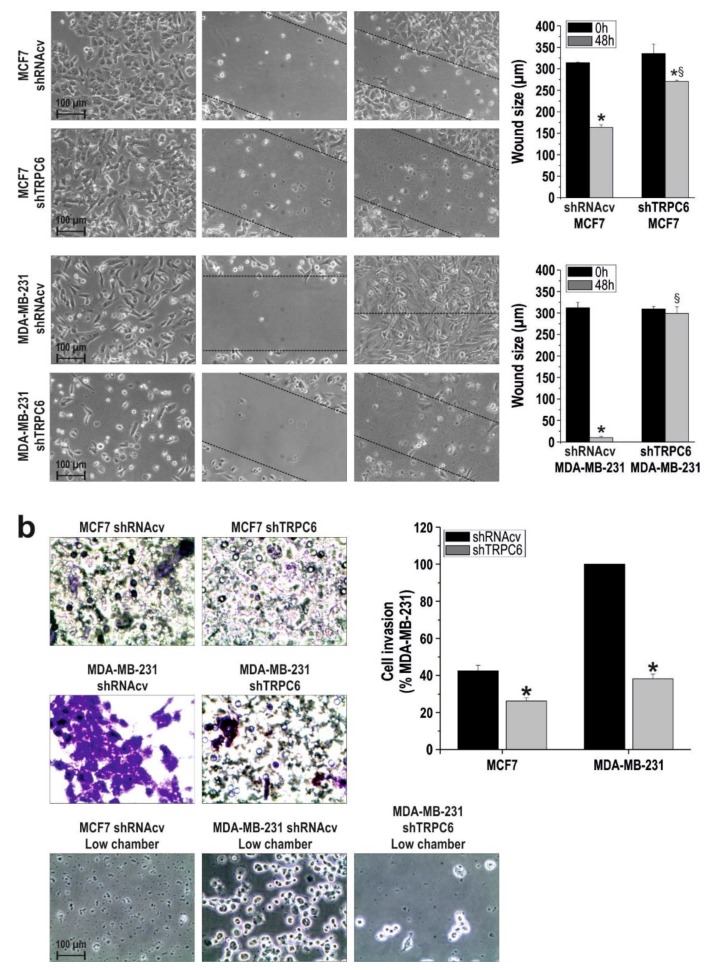
Role of TRPC6 in breast cancer cell migration and invasion. MCF10A, MCF7 and MDA-MB-231 cells were transfected with shTRPC6 or control shRNAcv. Forty-eight hours after transfection cells were subjected to wound healing assay (**a**) or transwell migration assay (**b**) as described in Methods. (**a**) Images were acquired at 0 and 48 h from the beginning of the assay. The dotted lines define the areas lacking cells. The bar graphs represent the wound size, in micrometers, at the different conditions, expressed as the mean ± SEM of three independent experiments. * *p* < 0.05 compared to the time = 0 h. § *p* < 0.05 compared to the corresponding time in shRNAcv transfected cells. (**b**) Images show the stained cells as obtained from the transwell migration assay subjected to the different experimental conditions. The bar graphs represent the percentage of cell invasion as compared to MDA-MB-231 cells transfected with shRNAcv, expressed as the mean ± SEM of five independent experiments. * *p* < 0.05 compared to the corresponding shRNAcv transfected cells. Bottom panels show representative pictures of the invasive cells adhered to the bottom of the lower chamber.

Next, we assessed the relevance of TRPC6 in the ability of these cell lines to migrate. MCF10A, MCF7 and MDA-MB-231 cells were subjected to the well-established wound healing assay. Cells were seeded, scratched, and cultured in medium supplemented with 1% serum to prevent further cell growth. Migration of cells was quantitated as described in Materials and Methods. To explore the role of TRPC6 in cell migration MCF10A, MCF7 and MDA-MB-231 cells were transfected with shTRPC6 or control plasmid and cell migration was evaluated. As shown in Figure 3a, MCF10A, MCF7 and MDA-MB-231 cells transfected with shRNAcv significantly reduced the wound size during the first 48 h (*p* < 0.05; n = 3). TRPC6 expression silencing did not affect the ability of MCF10A to migrate (Figure 3a; n = 3), which is consistent with the low expression of TRPC6 in this cell line. Interestingly, silencing TRPC6 expression significantly attenuated MCF7 and MDA-MB-231 migration as compared to cells transfected with shRNAcv (Figure 3a; *p* < 0.05; n = 3), which indicates that TRPC6 plays an important role in MCF7 and MDA-MB-231 cell migration.

We have further investigated the role of TRPC6 in in vitro invasion analysed using the transwell migration assay. After transfection with shRNAcv, a significant amount of MCF7 and MDA-MB-231 cells, especially the latter, passed across the transwell insert (Figure 3b). We even found a large number of MDA-MB-231 cells adhered to the surface of the lower chamber (Figure 3b, bottom panel). By contrast, we were unable to detect MCF10A cells in the undersurface of the transwell insert [32]. Interestingly, as depicted in Figure 3b, a lesser number of MCF7 and MDA-MB-231 cells were able to migrate to the undersurface of the transwell insert upon TRPC6 expression silencing as compared to cells treated with control shRNA (*p* < 0.05; n = 5). Consistently, the number of invasive MDA-MB-231 cells attached to the surface of the lower chamber was clearly reduced after transfection with shTRPC6 (Figure 3b, bottom panel). 

**Figure 4:**
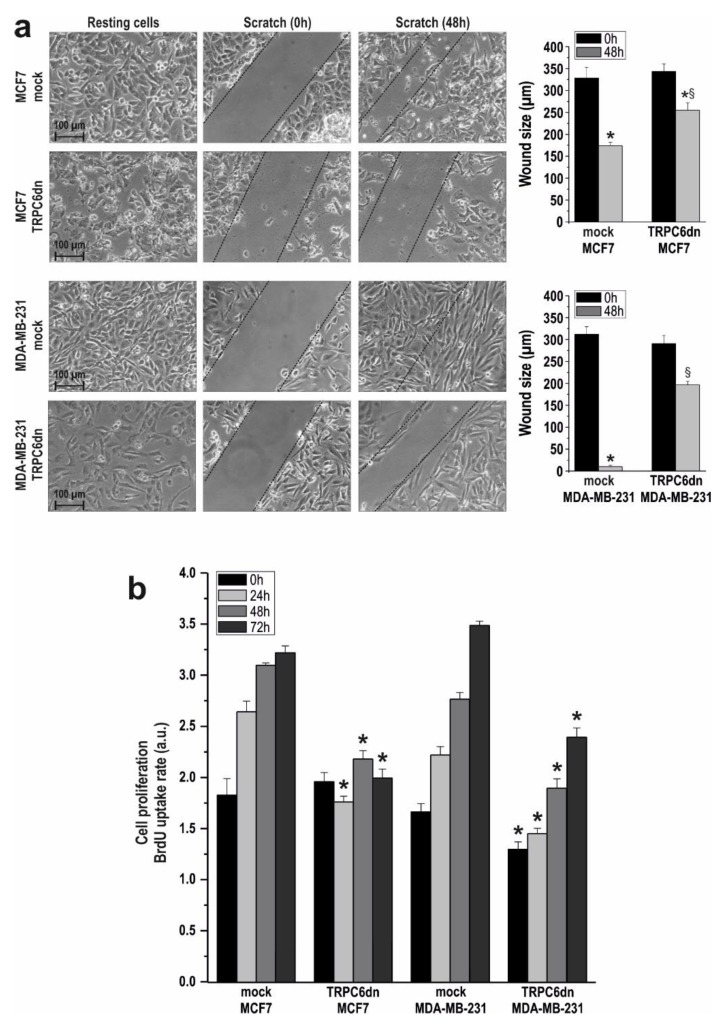
Expression of TRPC6dn mutant attenuates cell migration and proliferation in breast cancer cells. (**a**) MCF7 and MDA-MB-231 cells were transfected with TRPC6dn expression plasmid or empty vector (mock), as indicated. Forty-eight hours after transfection cells were subjected to wound healing assay as described in Methods. Images were acquired at 0 and 48 h from the beginning of the assay. The dotted lines define the areas lacking cells. The bar graphs represent the wound size, in micrometers, at the different conditions, expressed as the mean ± SEM of three independent experiments. * *p* < 0.05 compared to the time = 0 h. § *p* < 0.05 compared to the corresponding time in mock-treated cells. (**b**) MCF7 and MDA-MB-231 cells were transfected with TRPC6dn expression plasmid or empty vector (mock), as indicated, and 48 h later cell proliferation was assessed for a further 24, 48 and 72 h using the BrdU cell proliferation assay kit, as described in the Material and Methods. Bar graphs represent cell proliferation 0, 24, 48 and 72 h after cell transfection, presented as BrdU uptake rate. * *p* < 0.05 compared to the corresponding control (mock-transfected cells).

We confirmed the role of TRPC6 in breast cancer cell migration and proliferation by expressing a pore-dead dominant-negative TRPC6 (TRPC6dn) mutant. As shown in Figure 4a, expression of the TRPC6dn mutant significantly reduced MCF7 and MDA-MB-231 migration as compared to cells transfected with empty vector (*p* < 0.05; n = 3). 

Furthermore, expression of the TRPC6dn mutant significantly attenuated MCF7 and MDA-MB-231 cell proliferation at all the times investigated as compared to cells transfected with empty vector (Figure 4b; *p* < 0.05; n = 3). These findings confirm that TRPC6 is required for MCF7 and MDA-MB-231 breast cancer cells migration and proliferation.

### 2.2. Functional Role of TRPC6 in SOCE in Breast Cancer Cell Lines

**Figure 5:**
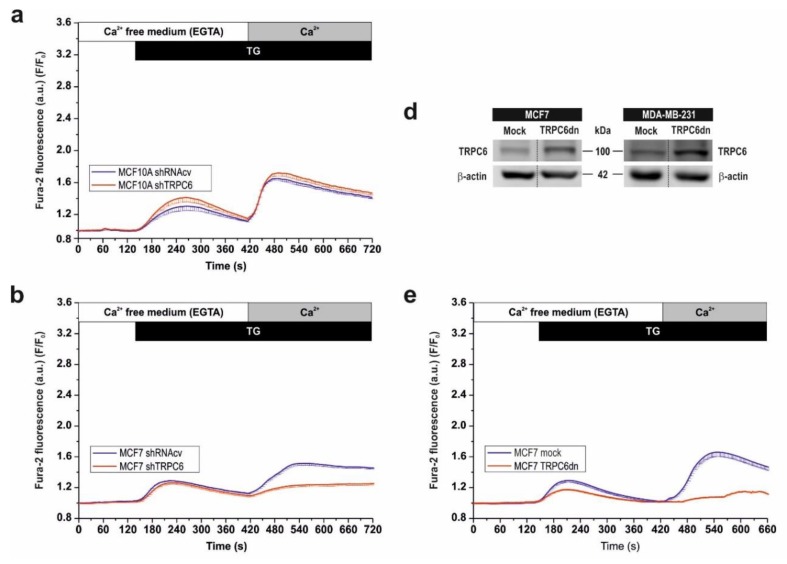

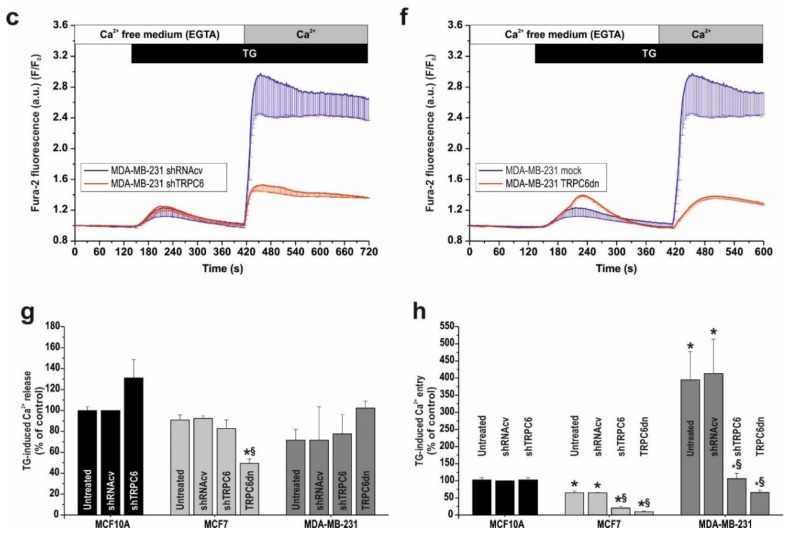
TRPC6 is required for store-operated Ca^2+^ entry in breast cancer cell lines. (**a**–**c**) MCF10A, MCF7 and MDA-MB-231 cells were transfected with shTRPC6 or scramble plasmid (shRNAcv), as indicated. Forty-eight hours after transfection, fura-2-loaded cells were perfused with a Ca^2+^-free medium (100 µM EGTA added) and then stimulated with TG (1 µM) followed by reintroduction of external Ca^2+^ (final concentration 1 mM) to initiate Ca^2+^ entry. Data are mean ± SEM of 40 cells/day/3–5 days. (**d**–**f**) MCF7 and MDA-MB-231 cells were transfected with TRPC6dn mutant expression plasmid or empty vector (mock), as indicated. After 48 h cells were lysed and subjected to western blotting with anti-TRPPC6 antibody, followed by reprobing with anti-β-actin antibody for protein loading control (**d**). Molecular masses indicated on the right were determined using molecular-mass markers run in the same gel. (**e** and **f**) Forty-eight hours after transfection, fura-2-loaded cells were perfused with a Ca^2+^-free medium (100 µM EGTA added) and then stimulated with TG (1 µM) followed by reintroduction of external Ca^2+^ (final concentration 1 mM) to initiate Ca^2+^ entry. Data are mean ± SEM of 40 cells/day/3–5 days. Bar graphs represent TG-induced Ca^2+^ release (**g**) and entry (**h**) in MCF10A, MCF7 and MDA-MB-231 cells untreated or transfected with the indicated plasmids. Data are expressed as mean ± SEM and presented as percentage of control (MCF10A cells treated with scramble plasmid). * represents *p* < 0.05 as compared to scramble-treated MCF10A cells. § represents *p* < 0.05 as compared to the same cell line transfected with shRNAcv.

As our results indicate that TRPC6 knockdown significantly attenuates relevant features of cancer cells, such as proliferation, migration and in vitro invasion, we have explored the possible mechanism underlying the functional role of TRPC6 in these cells. SOCE has been reported to play an important role supporting several cancer hallmarks [16,33,34]. Hence, we have evaluated whether TRPC6 plays a role in the activation of SOCE in breast cancer cells by transfecting non-tumoral MCF10A and cancer MCF7 and MDA-MB-231 cells with shTRPC6 or shRNAcv, as control. As depicted in Figure 5a–c, in cells transfected with shRNAcv suspended in a Ca^2+^-free medium, treatment with the SERCA inhibitor TG (1 µM) resulted in a transient increase in cytosolic free-Ca^2+^ concentration due to Ca^2+^ release from the intracellular Ca^2+^ stores. Subsequent addition of CaCl_2_ (1 mM) to the extracellular medium resulted in a further increase in cytosolic free-Ca^2+^ concentration indicative of SOCE. TG-induced Ca^2+^ release was similar in all the cell lines investigated while Ca^2+^ influx was significantly greater in MDA-MB-231 cells (Figure 5g,h; *p* < 0.05; n = 40 cells/day/3–5 days). Attenuation of TRPC6 expression by cell transfection with shTRPC6 significantly inhibited SOCE in MCF7 and MDA-MB-231 cells by 70%, without having any effect on Ca^2+^ release from the intracellular stores (Figure 5a–c,g–h; *p* < 0.05). Transfection of MCF10A cells with shTRPC6 did not significantly alter TG-induced Ca^2+^ release or entry, which is consistent with the low TRPC6 expression at the protein level in these cells. Altogether these findings indicate that TRPC6 plays a relevant role in the activation of SOCE in MCF7 and MDA-MB-231 breast cancer cells while this protein has not a detectable role in non-tumoral MCF10A cells.

In order to further explore whether the observed effect depends on cation entry through the channel or it is rather associated to a mechanism involving the expression of the protein itself, we overexpressed the TRPC6dn mutant in MCF7 and MDA-MB-231 cells and looked for its effect on TG-induced Ca^2+^ release and entry. As shown in Figure 5d, TRPC6dn was efficiently expressed in both cell types. As depicted in Figure 5e–h, overexpression of TRPC6dn in MCF7 and MDA-MB-231 cells significantly reduced TG-evoked Ca^2+^ entry to a similar extent to transfection of shTRPC6 (*p* < 0.05 as compared to control; n = 40 cells/day/3–5 days), which indicates that cation influx through TRPC6 plays an important role in SOCE in these cells. Overexpression of TRPC6dn also resulted in a significant decrease in the ability of MCF7 cells to accumulate Ca^2+^ into TG-sensitive stores (Figure 5e,g; *p* < 0.05; n = 40 cells/day/3–5 days), an effect that might be attributed to the inhibition of SOCE.

### 2.3. TRPC6 Expression Is Required for Plasma Membrane Localization of Orai1 and Orai3 in Breast Cancer Cells

**Figure 6:**
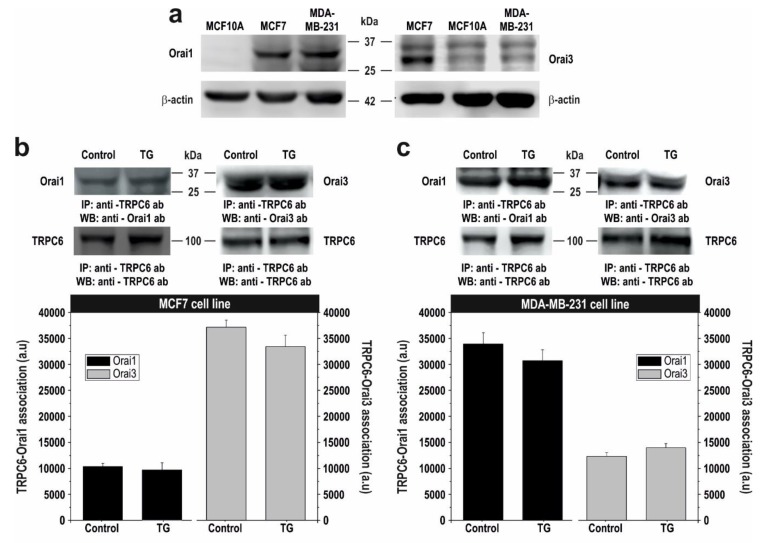

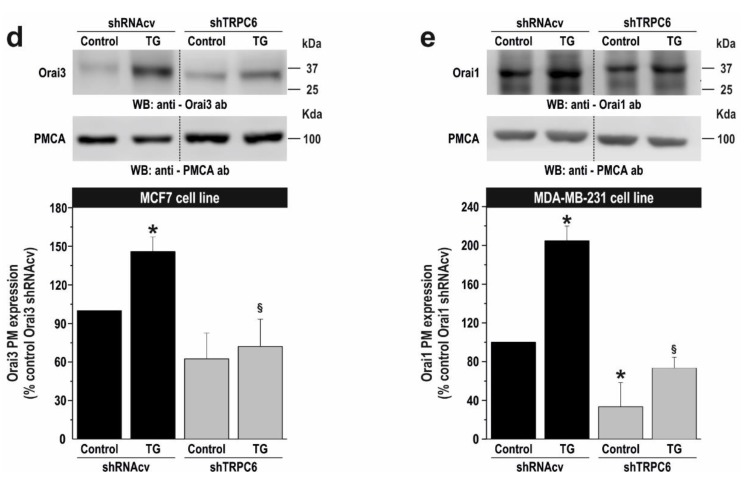
TRPC6 modulates plasma membrane localization of Orai1 and Orai3 in MDA-MB-231 and MCF7 breast cancer cells. (**a**) MCF10A, MCF7 and MDA-MB-231 cells were lysed and subjected to western blotting with anti-Orai1 or anti-Orai3 antibody, as indicated, followed by reprobing with anti-β-actin antibody for protein loading control. (**b,c**) MCF7 (**b**) and MDA-MB-231 (**c**) cells were left untreated or stimulated with TG (1 µM) for 3 min, lysed and whole cell lysates were immunoprecipitated (IP) with anti-TRPC6 antibody. Immunoprecipitates were subjected to 10% SDS-PAGE and subsequent western blotting with specific anti-Orai1 or anti-Orai3 antibody, as indicated. Membranes were reprobed with the antibody used for immunoprecipitation for protein loading control. The panels show results from one experiment representative of five others. Molecular masses indicated on the right were determined using molecular-mass markers run in the same gel. Bar graphs represent the quantification of TRPC6-Orai1 and TRPC6-Orai3 interaction in resting (control) and TG-treated cells. Results are presented as arbitrary optical density units, expressed as mean ± S.E.M. (**d**,**e**) MCF7 (**d**) and MDA-MB-231 cells (**e**) were transfected with shTRPC6 or scramble plasmid (shRNAcv), as indicated. Forty-eight hours after transfection, cells were stimulated with 1 µM TG in a medium containing 1 mM Ca^2+^, as indicated, and plasma membrane resident proteins were labeled by biotinylation, as described under Material and Methods. The biotinylated fraction was separated in 10% SDS-PAGE and analyzed by western blotting using either anti-Orai1 or anti-Orai3 antibody, as indicated. Membranes were reprobed with anti-PMCA antibody, as control. Positions of molecular mass markers are shown on the right. These results are representative of four separate experiments. Bar graphs represent the quantification of Orai3 (**d**) and Orai1 (**e**) surface exposition. Results are recorded as arbitrary optical density units, expressed as mean ± S.E.M. and presented as percentage of control (resting cells). * *p* < 0.05 as compared to resting cells transfected with shRNAcv. § *p* < 0.05 as compared to TG-treated cells transfected with shRNAcv.

Breast cancer MCF7 and MDA-MB-231 cells have been reported to express both Orai1 and Orai3 channels. However, the relative expression level and function differs from ER^+^ MCF7 cells to triple negative MDA-MB-231 cells [35]. While SOCE in MDA-MB-231 cells entirely depends on Orai1, MCF7 SOCE is mainly mediated by Orai3, whose expression, regulated by ERα [17], is predominant over that of Orai1 [35]. Our results confirm that Orai1 is overexpressed in the breast cancer cell lines and that Orai3 expression is significantly enhanced in MCF7 (Figure 6a; *p* < 0.05; n = 6), as previously reported [35]. In order to explore the mechanism underlying the sensitivity of SOCE to TRPC6 expression and function we have first investigated the interaction of TRPC6 with Orai1 and Orai3 by co-immunoprecipitation from MCF7 and MDA-MB-231 cell lysates. Resting and TG-treated cells were used for this study to determine whether Ca^2+^ store depletion plays any role in the possible interaction between TRPC6 and the Orai proteins investigated. As shown in Figure 6b,c, immunoprecipitation of cell lysates with anti-TRPC6 antibody followed by Western blotting with anti-Orai1 or anti-Orai3 antibody reveals that TRPC6 interacts with both proteins in resting cells. Interestingly, our results suggest that in MCF7 cells the interaction of TRPC6 with Orai3 is apparently greater than with Orai1, and, conversely, in MDA-MB-231 cells, TRPC6 seems to interact predominantly with Orai1 over Orai3 (Figure 6b,c; n = 6). Although these apparent differences might be attributed to the use of two different antibodies, if we look at the association of TRPC6 with Orai1, whose expression we and others have found to be similar in MCF7 and MDA-MB-231 cells [35] (Figure 6a), and we normalize the data with the amount of TRPC6 pulled down, our results indicate that more Orai1 is bound to TRPC6 in MDA-MB-231 cells (*p* < 0.05; n = 6). In addition, we have found that the interaction of TRPC6 with Orai1 and Orai3 is not altered by treatment with 1 µM TG for 3 min (Figure 6b,c), which, as depicted in Figure 5, is able to induce significant store depletion.

Similar results were obtained when cell lysates were immunoprecipitated with anti-Orai1 or anti-Orai3 antibody followed by western blotting with anti-TRPC6 antibody (Figure S1). These findings indicate that the interaction of TRPC6 with Orai1 and Orai3 is constitutive and not modified by Ca^2+^ store depletion.

We have further explored the role of cation influx by TRPC6 on the interaction between TRPC6 with Orai1 in MDA-MB-231 cells and Orai3 in MCF7 cells by expressing the pore-dead TRPC6dn mutant. As shown in Figure S2, expression of the TRPC6dn significantly attenuated the interaction of TRPC6 with the Orai channels in MCF7 and MDA-MB-231 cells (*p* < 0.05; n = 4), thus suggesting that TRPC6 channel function is essential for its interaction with Orai3 in MCF7 and Orai1 in MDA-MB-231 breast cancer cells.

Orai1 and Orai3 have been reported to account for most of the Ca^2+^ influx during the activation of SOCE in MDA-MB-231 and MCF7 cells, respectively [35], and our results indicate that TRPC6 knockdown results in similar attenuation of Ca^2+^ influx to that previously reported after Orai1 and Orai3 knockdown [35]. Hence, it is quite unlikely that TRPC6 and either Orai1 or Orai3 operate in separate pathways. A possible explanation for SOCE dependency on TRPC6 channel is that attenuation of TRPC6 expression reduces the plasma membrane localization of Orai1 and Orai3 in MDA-MB-231 and MCF7, respectively, where these channels have been found to be essential for SOCE [17,33,35]. Thus, we analysed the plasma membrane localization of Orai1 in MDA-MB-231 cells and Orai3 in MCF7 cells in cells transfected with shTRPC6 or shRNAcv, as control, by surface biotinylation. As shown in Figure 6d,e, surface exposition of Orai3 and Orai1 was clearly detected in MCF7 and MDA-MB-231 cells transfected with shRNAcv, respectively, and the presence of both channels in the plasma membrane was significantly enhanced upon treatment with TG (*p* < 0.05; n = 6). Interestingly, silencing TRPC6 expression significantly attenuated resting and TG-stimulated Orai3 and Orai1 surface exposition in MCF7 and MDA-MB-231 cells, respectively (Figure 6d,e; *p* < 0.05; n = 6). By contrast, TRPC6 knockdown was without effect on the surface exposition of Orai1 in MCF7 and Orai3 in MDA-MB-231 cells (Figure S3). To exclude that the attenuated protein expression is attributed to a reduced overall expression we analysed the total amount of Orai1 and Orai3 in lysates of cells transfected with shTRPC6 or scramble plasmids. Our results indicate that silencing TRPC6 expression did not alter the expression of Orai1 or Orai3 proteins (Figure S4). Together, these findings suggest that TRPC6 is required for the plasma membrane localization of Orai1 and Orai3 in MDA-MB-231 and MCF7 cells, respectively.

## 3. Discussion

TRP channels have been reported to play important roles in physiological as well as pathological events. The TRP-dependent cation currents elicited by receptor stimulation, either involving Ca^2+^-dependent processes or membrane depolarization, have been found to be crucial for a wide range of cellular functions [36]. Furthermore, dysregulation of TRP channel function, mostly due to abnormal expression, mutations or anomalous subcellular location underlies the onset and progression of a variety of disorders, including cancer [37].

In breast cancer, TRPV4 plays a role in cell migration and metastasis via Ca^2+^-dependent remodeling of the actin cytoskeleton [38,39]. Furthermore, TRPM7 expression has been found to be correlated to metastasis as well as invasive breast cancer via activation of the MAPK pathway [40] and is required for MCF7 cell proliferation [41]. TRPV6 expression has been reported to be enhanced in ER- and HER2-positive breast cancer cells and is associated to cell migration and invasion in MDA-MB-231 cells [42]. Immunohistochemical analysis of 49 normal tissues and ductal breast carcinomas has revealed that TRPC6 is overexpressed in breast adenocarcinoma [43]. Furthermore, TRPC3, as well as TRPC6, are up-regulated in breast cancer biopsies and the breast cancer cell lines MCF7 and MDA-MB-231 cells [31]. In these cell lines, TRPC6 have been found to be required for cell growth [31]; however, the molecular basis of the functional role for TRPC6 in breast cancer cells remained unknown. The present study identifies TRPC6 as an ion channel that plays a relevant role supporting breast cancer cell proliferation, migration and invasion. As reported in normal and tumor breast tissues [43], we have found that TRPC6 expression is enhanced in ER^+^ and triple negative breast cancer cell lines as compared to non-tumoral breast cells. We have found that the functional role of TRPC6 in breast cancer cells is likely mediated by its regulatory role on the activation of SOCE, which is significantly attenuated in cells where TRPC6 expression had been reduced by transfection of specific shRNA as well as in cells overexpressing a pore-dead TRPC6 mutant. By contrast, TRPC6 expression silencing has a negligible effect, if any, in non-tumoral breast cells, which is consistent with the low TRPC6 expression in these cells.

**Figure 7:**
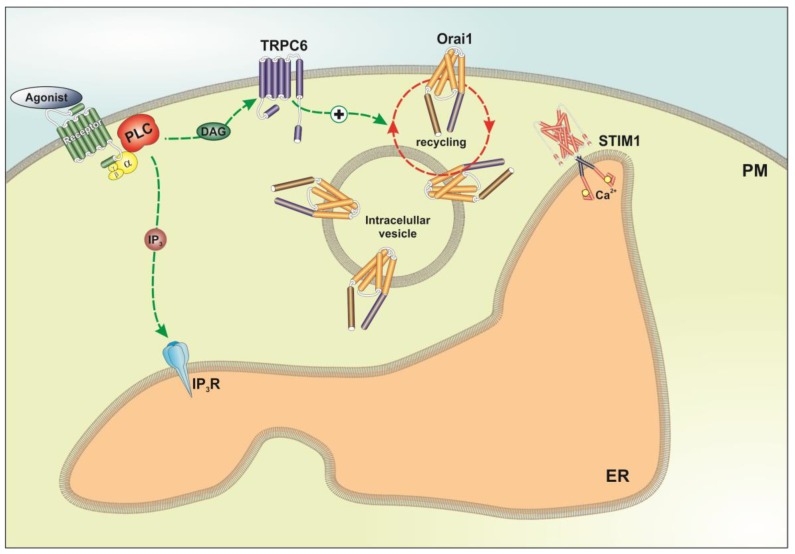
Proposed mechanism for the modulation of plasma membrane localization of Orai1 in MDA-MB-231 by TRPC6. Stimulation of MDA-MB-231 cells with Ca^2+^ mobilizing agonists might lead to phospholipase C (PLC) activation, which, in turn, results in the generation of IP_3_ and diacylglycerol (DAG). IP_3_ induces Ca^2+^ release from the ER while DAG results in the activation of TRPC6 channels (here only represented in the plasma membrane (PM) for simplicity). Ion influx via TRPC6 is required for the plasma membrane localization of Orai1, which, upon interaction with the ER Ca^2+^ sensor STIM1 participates in the activation and maintenance of SOCE in these cells. This molecular signalplex might play a functional role with relevance in cell proliferation and migration.

SOCE in MCF7 cells has been reported to be mostly dependent on STIM1, STIM2 and Orai3 [35], a channel that, in agreement with previous studies [35], we have found to be overexpressed in these cell line. On the other hand, SOCE in MDA-MB-231 cells is mostly mediated by STIM1 and Orai1 [35]. As SOCE in breast cancer cells depends on the Orai channels, and the extent of SOCE inhibition in cells transfected with shTRPC6 in our hands was similar to that reported by Motiani and coworkers after Orai1 and Orai3 knockdown in MDA-MB-231 and MCF7, respectively [35], we hypothesized that TRPC6 might be regulating the Orai channels rather than playing a major role in the conduction of Ca^2+^ entry during SOCE. TRP channels have been previously shown to modulate other ion channels in different ways. For instance, TRPA1 is a negative modulator of the STIM1-Orai1 interaction in megakaryoblastic cells [44], and TRPC1 is a suppressor of plasma membrane targeting of TRPV6 channels [45]. Based on the previously mentioned observations we further evaluated the possible role of TRPC6 in the surface exposition of Orai1 and Orai3 in MCF7 and MDA-MB-231 cells. Interestingly, we have found that TRPC6 is required for the specific plasma membrane localization of Orai3 in MCF7 and Orai1 in MDA-MB-231 cells, both at resting conditions and after stimulation with TG, within a molecular signalplex that modulates SOCE and cell function (Figure 7). On the other hand, the surface exposition of Orai1 in MCF7 or Orai3 in MDA-MB-231 cells were found to be independent of TRPC6 expression or Ca^2+^ store depletion. This latter finding confirms the results presented by Motiani and coworkers [35]. The regulation of Orai3 and Orai1 plasma membrane localization in MCF7 and MDA-MB-231 cells, respectively, by TRPC6 might explain the similar dependence of SOCE on the Orai and TRPC6 channels in these cell types. In summary, we provide strong evidence for a role of TRPC6 as a new regulator of SOCE, cell proliferation, migration and invasion in breast cancer cells.

## 4. Materials and Methods

### 4.1. Reagents

Fura-2 acetoxymethyl ester (fura-2/AM) was from Molecular Probes (Leiden, The Netherlands). Thapsigargin (TG), rabbit polyclonal anti-Orai1 antibody (catalog number O8264, epitope: amino acids 288–301 of human Orai1), mouse monoclonal anti-Orai3 antibody (clone 1B4F1, epitope: 19 amino acid peptide from near the C-terminus), rabbit polyclonal anti-β-actin antibody (catalog number A2066, epitope: amino acids 365–375 of human β-actin), and bovine serum albumin (BSA) were from Sigma (Madrid, Spain). Rabbit polyclonal anti-TRPC6 antibody (catalog number: ACC-120, epitope corresponding to amino acid residues 573–586) was from Alomone (Jerusalem, Israel). Turbofect transfection reagent, mouse monoclonal anti-PMCA antibody (Clone 5F10, epitope: amino acids 724–783 of human PMCA), EZ-Link Sulfo-NHSLC-Biotin and streptavidin–conjugated agarose beads were from Thermo Fisher (Madrid, Spain). Horseradish peroxidase-conjugated anti-mouse IgG antibody and anti-rabbit IgG antibody for IP (not recognizing the heavy and light chains of the immunoprecipitating antibody) were from Abcam (Cambridge, UK). shRNA control vector was from Origene (Rockville, MD, USA). Protein A-agarose was from Upstate Biotechnology Inc. (Madrid, Spain). Complete EDTA-free protease inhibitor tablets were from Roche (Madrid, Spain). Enhanced chemiluminescence detection reagents were from Pierce (Cheshire, UK). Bromodeoxyuridine (BrdU) cell proliferation assay kit was from BioVision (Milpitas, CA, USA). All other reagents were of analytical grade.

### 4.2. Cell Culture and Transfection

MCF10A were provided by Dr. Potier-Cartereau (Université François Rabelais Tours, France). MCF7 and MDA-MB-231 cell lines were obtained from ATCC (Manassas, VA, USA), and cultured at 37 °C with a 5% CO_2_ in DMEM-F12 (MCF10A) or DMEM (MCF7 and MDA-MB-231), supplemented with 10% (*v*/*v*) horse or fetal bovine serum, respectively, and 100 U/mL penicillin and streptomycin.

Cells were transfected with expression plasmids for the dominant-negative mutant of TRPC6 (TRPC6dn; kindly provided by Dr. Kristina Friedland), as well as with the shTRPC6 or scramble plasmids as described previously [46,47,48] using Turbofect transfection reagent and were used 48 h after transfection. Plasmids were used for silencing experiments at 1 µg/mL.

### 4.3. Measurement of Cytosolic Free-Calcium Concentration 

Cells were loaded with fura-2 by incubation with 2 μM fura 2/AM for 30 min at 37 °C. Coverslips with cultured cells were mounted on a perfusion chamber and placed on the stage of an epifluorescence inverted microscope (Nikon Eclipse Ti2, Amsterdam, The Netherlands) with image acquisition and analysis system for videomicroscopy (NIS-Elements Imaging Software, Nikon). Cells were continuously superfused with HEPES-buffered saline (HBS) containing (in mM): 125 NaCl, 5 KCl, 1 MgCl_2_, 5 glucose, 25 HEPES, and pH 7.4, supplemented with 0.1% (*w*/*v*) BSA. Cells were alternatively excited with light from a xenon lamp passed through a high-speed monochromator (Optoscan ELE 450, Cairn Research, Faversham, UK) at 340/380 nm. Fluorescence emission at 505 nm was detected using a cooled digital sCMOS camera (Zyla 4.2, Andor, Belfast, UK) and recorded using NIS-Elements AR software (Nikon, Amsterdam, The Netherlands). Fluorescence ratio (F340/F380) was calculated pixel by pixel, and the data are presented as F/F_0_, where F is the experimental fura-2 340/380 fluorescence ratio and F_0_ is the mean basal fura-2 340/380 fluorescence ratio [49]. TG-evoked Ca^2+^ release and influx was measured as the integral of the rise in ΔF/F_0_ for 2½ min after the addition of TG in the absence or presence of extracellular Ca^2+^, respectively.

### 4.4. Immunoprecipitation and Western Blotting

The immunoprecipitation and western blotting were performed as described previously [50]. Briefly, 500 µL aliquots of cell suspension (5 × 10^6^ cell/mL) were lysed with an equal volume of ice-cold 2 × NP-40 buffer, pH 8, containing 274 mM NaCl, 40 mM Tris, 4 mM EDTA, 20% glycerol, 2% nonidet P-40, 2 mM Na_3_VO_4_ and complete EDTA-free protease inhibitor tablets. Aliquots of cell lysates (1 mL) were immunoprecipitated by incubation with 1 µg of anti-TRPC6 antibody and 25 µL of protein A-agarose overnight at 4 °C on a rocking platform. The immunoprecipitates were resolved by 10% SDS-PAGE and separated proteins were electrophoretically transferred onto nitrocellulose membranes for subsequent probing. Blots were incubated overnight with 10% (*w*/*v*) BSA in tris-buffered saline with 0.1% Tween 20 (TBST) to block residual protein binding sites. Immunodetection of Orai1, Orai3, TRPC6, PMCA and β-actin was achieved by incubation for 2 h with anti-Orai1 antibody diluted 1:500 in TBST, overnight with anti-Orai3 or anti-PMCA antibody diluted 1:1000 in TBST, overnight with anti-TRPC6 antibody diluted 1:500 in TBST or for 1 h with anti- β-actin antibody diluted 1:2000 in TBST. The primary antibody was removed and blots were washed six times for 5 min each with TBST. To detect the primary antibody, blots were incubated for 1 h with horseradish peroxidase-conjugated goat anti-mouse IgG antibody or horseradish peroxidase-conjugated goat anti-rabbit IgG antibody diluted 1:10000 in TBST and then exposed to enhanced chemiluminiscence reagents for 5 min. The density of bands was measured using C-DiGit Chemiluminescent Western Blot Scanner (LI-COR Biosciences, Lincoln, NE, USA). Data were normalized to the amount of protein recovered by the antibody used for the immunoprecipitation.

### 4.5. Transwell Migration Assay

Migration assay was performed using cell culture inserts with 8 μm pores (BD Biosciences, Frankin Lakes, NJ, USA). Cell culture inserts were placed in the 24-well plate containing 750 μL of DMEM without serum (chemo-attractant). In the upper half of the insert 2.5 × 10^5^ cells were placed inside the chamber. DMEM containing 10% FBS was added to the lower chamber of the 24-well plate. After 24 h the cells in the insert were washed with PBS, fixed with formaldehyde (3.7%) and permeabilized using methanol. Cells were stained with Giemsa stain for 30 min. Cells present in the lower side of the inserts were counted in five microscopic fields per well, and the extent of migration was expressed as an average number of cells per microscopic field.

### 4.6. Wound Healing Assay

For wound healing assay, MCF7 and MDA-MB-231 cells were seeded in 35-mm 6 well multidish to obtain confluence after 24 h. Next, cells were cultured in medium supplemented with 1% serum and a wound was created using a sterile 200-µL plastic pipette tip. Photographs were taken immediately or at the times indicated using an inverted microscope (Nikon Eclipse TS100, Tokio, Japan). Migration of cells was quantitated using Fiji ImageJ (NIH, Bethesda, MD, USA).

### 4.7. Determination of Cell Proliferation

Cells were seeded at a concentration of 5 × 10^3^/well into 96-well plates and after 0, 24, 48 and 72 h, cell proliferation was assessed by a specific cell proliferation assay kit based on the measurement of BrdU incorporation during DNA synthesis according to the manufacturer’s instructions (BioVision). Absorbance in samples was measured using a plate reader (Epoch, Biotek, Swindon, UK) at 450 nm and presented as arbitrary units.

### 4.8. Biotinylation Protocol

Cells were washed 3 times with phosphate-buffered saline (PBS, NaCl 137 mM, KCl 2.7 mM, KH_2_PO_4_, 1.5 mM, Na_2_HPO_4_•2H_2_O 8mM, pH 7.4), subsequently resuspended in biotinylation buffer (50 mM NaHCO_3_ and 0.9% NaCl) and surface labeled with 100 mg/mL sulfo-NHS-LC biotin at RT. Labeling was stopped 1 h after reaction with 1% NH_4_Cl and PBS supplemented with 50 mM EDTA and washed 2 times in PBS/EDTA. Biotinylated cells were subsequently lysed in Nonidet P40 buffer (NP40), and protein lysates were incubated with streptavidin-conjugated agarose beads overnight at 4 °C on a rocking platform. Biotinylated proteins bound to streptavidin-conjugated agarose beads were isolated by centrifugation and washed 3 times in NP40 buffer. Biotinylated fraction was loaded and separated in 10% SDS-PAGE and analyzed by western blotting using anti-PMCA antibody, as control, or either anti-Orai1 or anti-Orai3 antibody [51].

### 4.9. Statistical Analysis

Analysis of statistical significance was performed using one-way analysis of variance. For comparison between two groups Student’s *t* test was used. *p* < 0.05 was considered to be significant for a difference.

## 5. Conclusions

The present study demonstrates that TRPC6 plays an important functional role supporting a variety of breast cancer hallmarks, including proliferation, migration and “in vitro” invasion. Our results indicate that TRPC6 expression is up-regulated in ER^+^ and triple negative breast cancer cell lines, where TRPC6 interacts with the most prominent Orai isoform for SOCE, Orai1 in MDA-MB-231 cells and Orai3 in MCF7, as previously described [35]. TRPC6 is required for the plasma membrane localization of Orai3 in MCF7 and Orai1 in MDA-MB-231 cells, which is essential for the activation of SOCE and cell function.

## Supplementary Materials

The following are available online at http://www.mdpi.com/2072-6694/10/9/331/s1, Figure S1: TRPC6 co-immunoprecipitates with Orai1 and Orai3 in MCF7 and MDA-MB-231 breast cancer cells. Figure S2: Expression of a TRPC6dn mutant impairs the interaction of TRPC6 with Orai channels in MCF7 and MDA-MB-231 breast cancer cells. Figure S3: TRPC6 knockdown does not alter the surface exposition of Orai1 and Orai3 in MDA-MB-231 and MCF7 breast cancer cells. Figure S4: TRPC6 knockdown does not modify Orai1 and Orai3 protein expression in non-tumoral and breast cancer cells.
