# Supplementary material for: TRPC6 Channels Are Required for Proliferation, Migration and Invasion of Breast Cancer Cell Lines by Modulation of Orai1 and Orai3 Surface Exposure
Source: Cancers (Basel). 2018 Sep 14;10(9):331. doi: 10.3390/cancers10090331 (PMC6162527; doi:10.3390/cancers10090331)
Supplement: Supplementary file 1 [file cancers-10-00331-s001.pdf]

Supplementary Materials

TRPC6 Channels Are Required for Proliferation, Migration and Invasion of Breast Cancer Cell Lines by Modulation of Orai1 and Orai3 Surface Exposure

Isaac Jardin, Raquel Diez-Bello, Jose J. Lopez, Pedro C. Redondo, Ginés M. Salido, Tarik Smani and Juan A. Rosado

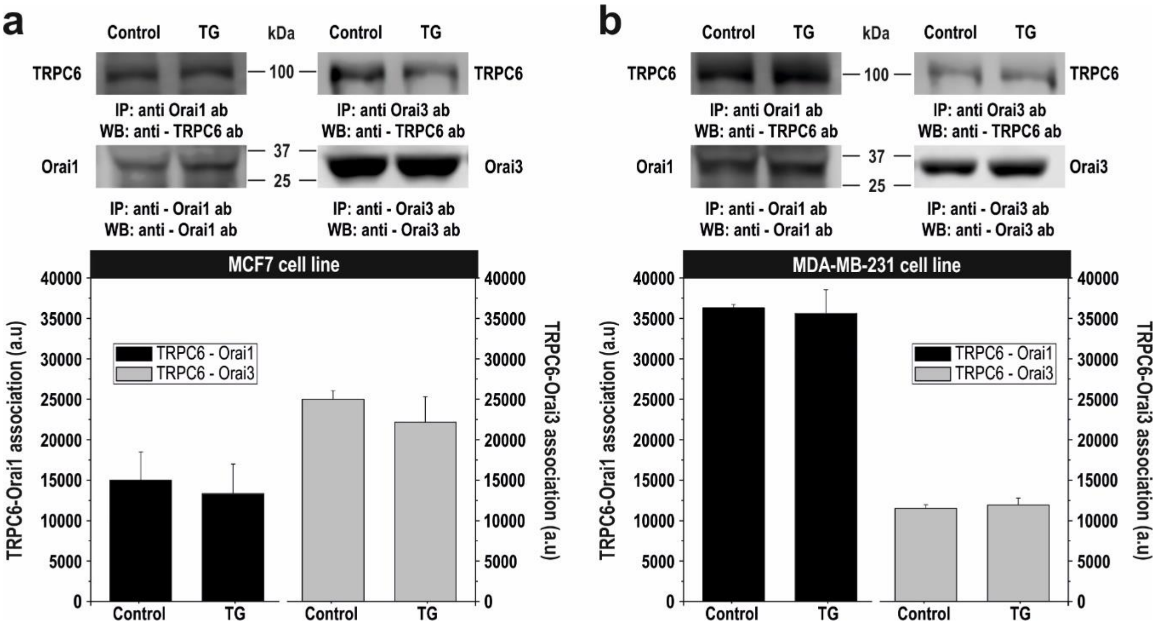

Figure S1. TRPC6 interacts with Orai1 and Orai3 in MCF7 and MDA-MB-231 breast cancer cells. MCF7 (**a**) and MDA-MB-231 (**b**) cells were left untreated or stimulated with TG (1 μM) for 3 min, lysed and whole cell lysates were immunoprecipitated (IP) with anti-Orai1 or anti-Orai3 antibody, as indicated. Immunoprecipitates were subjected to 10% SDS-PAGE and subsequent Western blotting with specific anti-TRPC6 antibody. Membranes were reprobed with the antibody used for immunoprecipitation for protein loading control. The panels show results from one experiment representative of five others. Molecular masses indicated on the right were determined using molecular-mass markers run in the same gel. Bar graphs represent the quantification of Orai1-TRPC6 and Orai3-TRPC6 interaction in resting (control) and TG-treated cells. Results are presented as arbitrary optical density units, expressed as mean ± S.E.M.

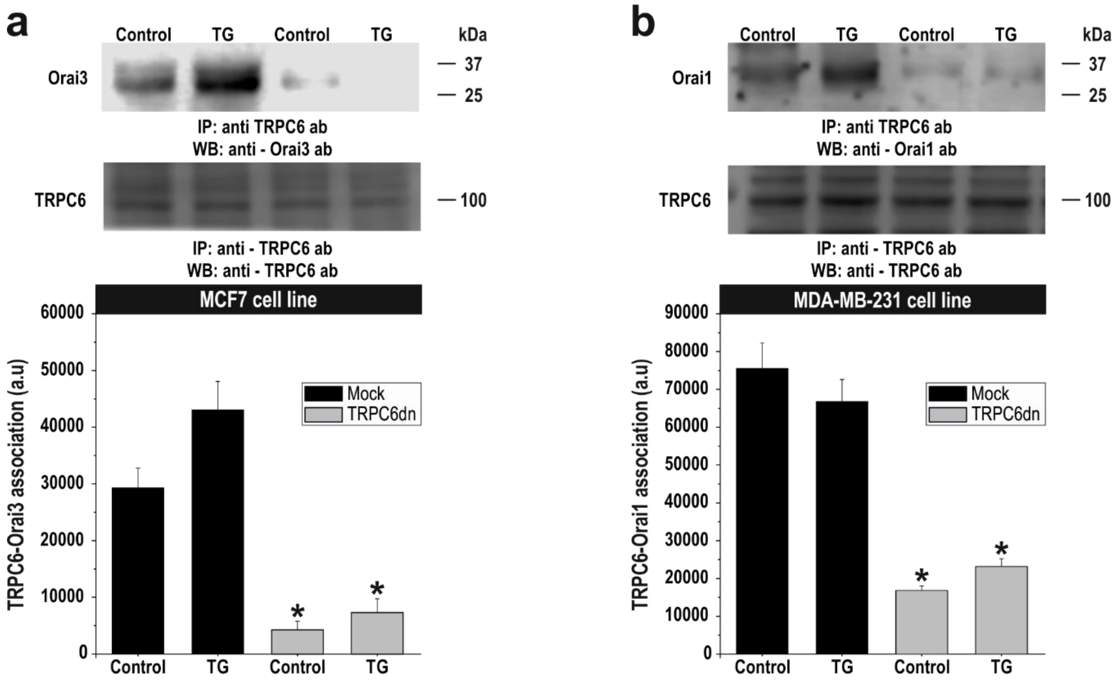

Figure S2. Expression of a TRPC6dn mutant impairs the interaction of TRPC6 with Orai channels in MCF7 and MDA-MB-231 breast cancer cells. MCF7 (**a**) and MDA-MB-231 (**b**) cells were transfected with TRPC6 dominant-negative (TRPC6dn) expression plasmid or empty vector (mock), as indicated. Forty-eight hours later cells were left untreated or stimulated with TG (1 μM) for 3 min, lysed and whole cell lysates were immunoprecipitated (IP) with TRPC6 antibody, as indicated. Immunoprecipitates were subjected to 10% SDS-PAGE and subsequent Western blotting with specific anti-Orai3 (for MCF7 cells) or anti-Orai1 (for MDA-MB-231 cells) antibody. Membranes were reprobed with the antibody used for immunoprecipitation for protein loading control. The panels show results from one experiment representative of three others. Molecular masses indicated on the right were determined using molecular-mass markers run in the same gel. Bar graphs represent the quantification of Orai3-TRPC6 (**a**) and Orai1-TRPC6 (**b**) interaction in resting (control) and TG-treated cells. Results are presented as arbitrary optical density units, expressed as mean ± S.E.M.

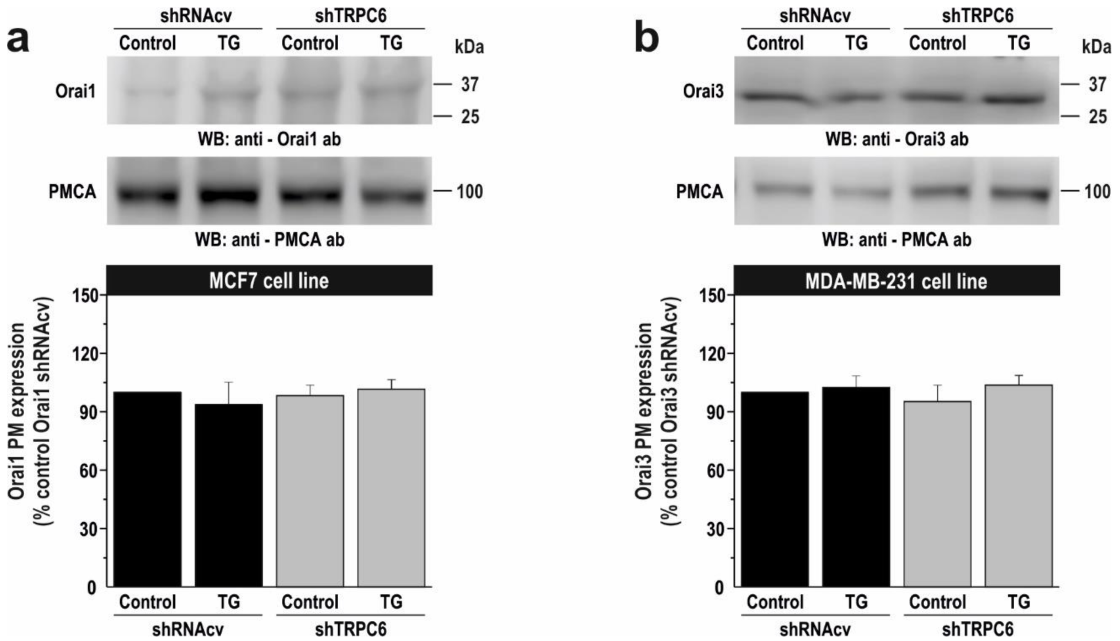

Figure S3. TRPC6 knockdown does not alter the surface exposition of Orai1 and Orai3 in MCF7 and MDA-MB-231 breast cancer cells. MCF7 (**a**) and MDA-MB-231 cells (**b**) were transfected with shTRPC6 or scramble plasmid (shRNAcv), as indicated. Forty-eight hours after transfection, cells

were stimulated with 1 μM TG in a medium containing 1 mM Ca^{2+}, as indicated, and plasma membrane proteins were labeled by biotinylation, as described under Material and Methods. The biotinylated fraction was separated in 10% SDS-PAGE and analyzed by Western blotting using either anti-Orai1 or anti-Orai3 antibody, as indicated. Membranes were reprobed with anti-PMCA antibody, as control. Positions of molecular mass markers are shown on the right. These results are representative of four separate experiments. Bar graphs represent the quantification of Orai1 (**a**) and Orai3 (**b**) surface exposition. Results are recorded as arbitrary optical density units, expressed as mean ± S.E.M. and presented as percentage of control (resting cells).

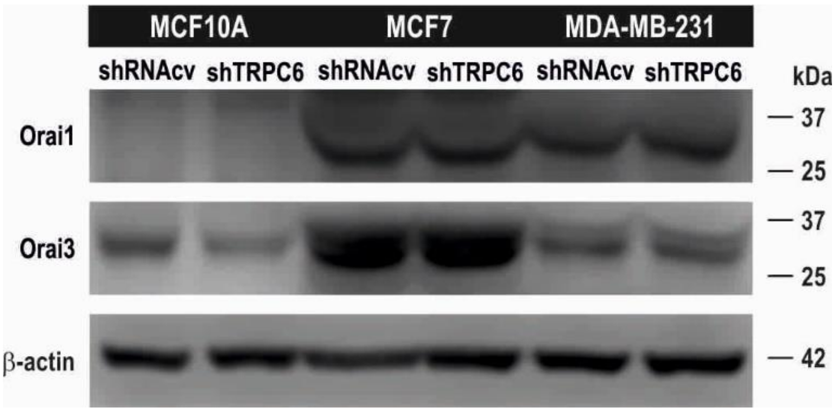

Figure S4. TRPC6 knockdown does not modify Orai1 and Orai3 protein expression in non-tumoral and breast cancer cells. MCF10A, MCF7 and MDA-MB-231 cells were transfected with shTRPC6 or scramble plasmid (shRNAcv), as indicated. Forty-eight hours after transfection, cells were lysed and subjected to Western blotting with anti-Orai1 or anti-Orai3 antibody, as indicated, followed by reprobing with anti-β-actin antibody for protein loading control. Molecular masses indicated on the right were determined using molecular-mass markers run in the same gel.
